# A curated collection of tissue microarray images and clinical outcome data of prostate cancer patients

**DOI:** 10.1038/sdata.2017.14

**Published:** 2017-03-14

**Authors:** Qing Zhong, Tiannan Guo, Markus Rechsteiner, Jan H. Rüschoff, Niels Rupp, Christian Fankhauser, Karim Saba, Ashkan Mortezavi, Cédric Poyet, Thomas Hermanns, Yi Zhu, Holger Moch, Ruedi Aebersold, Peter J. Wild

**Affiliations:** 1 Department of Pathology and Molecular Pathology, University Hospital Zurich, 8091 Zurich, Switzerland; 2 Department of Biology, Institute of Molecular Systems Biology, ETH Zurich, 8093 Zurich, Switzerland; 3 Department of Urology, University Hospital Zurich, 8091 Zurich, Switzerland; 4 Faculty of Science, University of Zurich, 8057 Zurich, Switzerland; 5 University of Zurich, 8006 Zurich, Switzerland

**Keywords:** Tumour heterogeneity, Prostate cancer, Outcomes research, Computational models

## Abstract

Microscopy image data of human cancers provide detailed phenotypes of spatially and morphologically intact tissues at single-cell resolution, thus complementing large-scale molecular analyses, e.g., next generation sequencing or proteomic profiling. Here we describe a high-resolution tissue microarray (TMA) image dataset from a cohort of 71 prostate tissue samples, which was hybridized with bright-field dual colour chromogenic and silver in situ hybridization probes for the tumour suppressor gene *PTEN*. These tissue samples were digitized and supplemented with expert annotations, clinical information, statistical models of *PTEN* genetic status, and computer source codes. For validation, we constructed an additional TMA dataset for 424 prostate tissues, hybridized with FISH probes for *PTEN*, and performed survival analysis on a subset of 339 radical prostatectomy specimens with overall, disease-specific and recurrence-free survival (maximum 167 months). For application, we further produced 6,036 image patches derived from two whole slides. Our curated collection of prostate cancer data sets provides reuse potential for both biomedical and computational studies.

## Background & Summary

Technical advances of large-scale molecular studies, including next generation genomic analyses and proteomic profiling, of human tissue samples have enabled discovery of genetic and other molecular aberrations in different regions of a tumour, defined as intra-tumour heterogeneity (ITH), having critical implications in precise diagnosis and treatment of cancers^1–5^. Yet, such studies often evaluate samples prepared from homogenised tissues and exclude corresponding histo-morphology, thereby failing to investigate molecular changes and to identify minor sub-clones at single-cell level.

We have recently developed an integrative method (ISHProfiler), combining an image-based computational workflow with a dual-colour chromogenic and silver in situ hybridization assay (DISH) that accurately detects copy number variation (CNV) with preserved histo-morphology at single-cell resolution, expressively visualizes multi-level heterogeneity (cellular, inter-, and intra-tumour heterogeneity), and objectively quantifies heterogeneous allelic gains and losses of various genes in diverse human tumours hybridized with molecular probes^6^. Our ISHProfiler supports broad applications in biomedical and computational research and alleviates the limitations of the gold standard method, fluorescence in situ hybridization (FISH)^7^. These include error-prone manual counting under a fluorescence microscope, severe inter-observer variability, and qualitative assessment of genetic status^8–10^.

**Figure 1:**
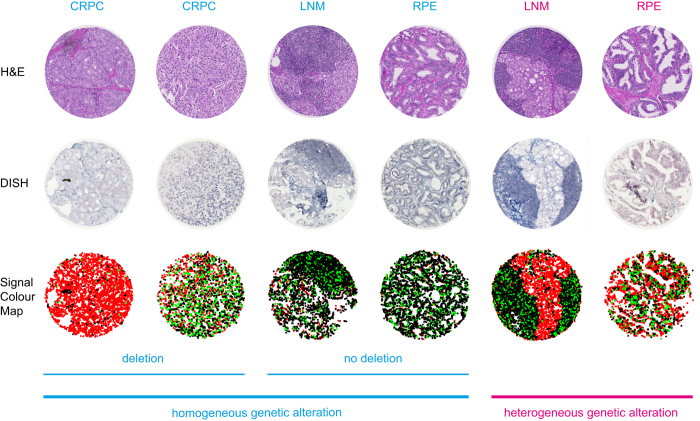
Sample tissue cores of matching H&E images, DISH images, and signal colour maps. Six examples of H&E images (top row), DISH images (middle row), and signal colour maps (bottom row) with patient-level expert annotations, such as tissue types and *PTEN* genetic status. Signal colour maps can be generated by our ISHProfiler software. For the signal colour maps, black signals indicate the *PTEN* gene, red signals indicate the CEP10, and green signal indicate the mixed classes *PTEN*+CEP10. CRPC, castration resistant prostate cancer; LNM, lymph node metastases; RPE, radical prostatectomy.

To benchmark the ISHProfiler, we have analysed a large number of stained tissue microarray (TMA) images with associated clinical data^6^. Here, we provide a more detailed description of these data and guarantee their open access for future data re-mining studies. Our collection of benign and malignant prostate formalin-fixed, paraffin-embedded (FFPE) tissue samples consist of *PTEN* DISH images of a TMA with corresponding signal colour maps of the *PTEN* gene and the corresponding centromeric probe (CEP) of chromosome 10 (*n*=71; Fig. 1), matching hematoxylin and eosin (H&E) images from the serial sections (*n*=71), patches of two whole slide *PTEN* DISH images (*n*=3,726 and *n*=2,310), patient-level annotations (*n*=71), clinical information (*n*=424), survival data (*n*=339), computational models (*n*=71), and computer source codes (Table 1). The 424 prostate FFPE tissue samples comprises 339 radical prostatectomy (RPE) specimens, 28 castration resistant prostate cancers (CRPCs), 17 lymph node metastases (LNM), 11 distant metastases (DM), and 29 benign prostatic hyperplasias (BPHs). In addition, the survival data exhibit a median follow-up of 95 months and a maximum of 167 months for the 339 RPE samples with additional clinico-pathological, immunological and molecular data (Table 2).

In comparison to comprehensive data collections such as TCGA^4^, CAMELYON16 (camelyon16.grand-challenge.org), TUPAC16 (www.tupac.tue-image.nl), and HER2 scoring contest (warwick.ac.uk/fac/sci/dcs/research/combi/research/bic/her2contest), our data resource is small but well curated. It combines images, genetic information, and the clinical data, into a unified computational model that quantifies *PTEN* genetic status as distribution. The data reveals multi-level tumour heterogeneity and alleviates the problem that genetic status is traditionally a binary classification. A potential reuse of this collection of data will be the investigation of whether a quantitative, model-based description of a heterogeneous genetic status is superior compared to a binary decision, when associating the results with clinical outcomes.

Although our related work^6^ quantifies the genetic alteration and tumour heterogeneity by classifying molecular signals of interest without consideration of tumour cell recognition^11,12^, the digitized DISH images retain intact tissue morphology, thus potentially enabling studies that address the detection of tumorous tissues and the re-analysis of genetic aberrations at single-cell resolution.

With patient-level annotation of *PTEN* genetic status of the 71 prostate cancer TMA samples acquired by two different scanning protocols, computational scientists can reuse the dataset for testing whether molecular signals such as genes of interests and the corresponding CEP can be recognized in an unsupervised fashion or independently of scanning procedures, thus completely avoiding the labour-intensive, error-prone, and subjective manual annotation of these molecular signals.

Selection of tumour tissue regions for high-throughput molecular profiling, such as genomic and proteomic studies, is currently accomplished by staining whole slide tissues with H&E, immnuhistochemistry (IHC) or in situ hybridization (ISH), followed by manual evaluation of small selected regions of interest by trained pathologists^6^. Our quantitative signal colour map produced by ISHProfiler, which preserves tissue topology and combines genetic analysis with clinico-pathological assessment, offers an alternative approach for hotspot tissue region selection from heterogeneous tumour tissues with a high degree of accuracy, objectivity and reproducibility.

## Methods

The following methods are either modified, shortened or expanded versions of the methods and Supplementary Information in our related work^6^.

### Prostate cancer patients

A total of 424 FFPE tissue samples were retrieved from the archives of the Department of Pathology and Molecular Pathology, University Hospital Zurich, Switzerland^13–17^. One tissue core (diameter 0.6 mm and thickness of 4 μm) of a representative tumour area per patient was taken from a ‘donor’ block and was arranged in a new ‘recipient’ block using a customized instrument. The TMA included a series of consecutive (non-selected) RPE specimens with localized prostate cancer, CRPCs, LNM, DM and BPHs. H&E-stained slides of all specimens were evaluated by two experienced pathologists to identify representative areas for TMA construction (H.M., P.J.W.). Specimens were annotated with clinical information such as patient demographics, histological findings, treatment, and outcome data including overall and disease-specific survival as well as biochemical (PSA) recurrence. Tumour stage and Gleason score of the cohort were assigned according to the International Union Against Cancer (UICC) and WHO/ISUP 2016 criteria. Gleason scores were assigned by two independent investigators (P.J.W., H.M.) and a consensus was achieved in case of discrepant results by both investigators. Effectively, 424 samples were used in the FISH analysis and a subset of 71 samples was used for DISH manual assessment and computational analysis (Data Citation 1 – Data Citation 3). The DISH subset comprises 38 primary acinar adenocarcinomas from RPE, 10 CRPCs, six PC LNM, one DM, and 16 BPHs. The study was approved by the Cantonal Ethics Committee of Zurich (StV-No. 2008-0025) and the associated methods were carried out in accordance with the approved guidelines.

### *PTEN* FISH analysis

For *PTEN* deletion analysis, a dual-colour FISH was performed using commercially available DNA probes for the region 10q23.3 (Spectrum Orange, *PTEN* locus-specific probe; Abbott Molecular) and 10p11.1-q11.1 (Spectrum Green, CEP of chromosome 10; LSI *PTEN*/CEP10; Abbott Molecular), as described previously^10^. In detail, four micron thick sections were deparaffinized in xylene before immersion in 100% ethanol. Sections were then placed in 10 mM citrate buffer (pH 6.0) at 96 °C for 15 min, followed by treatment with pepsin (Medite GmbH) at 37 °C for 40 min. Sections were dehydrated in a graded series of ethanol. Probes and target DNA were co-denatured at 75 °C for 10 min. Post-hybridization washings were performed with 2x SSC solution at room temperature and 73 °C for 2 min. Slides were then air-dried in dark. Nuclei were counterstained with 4′,6-diamidino-2-phenylindole (DAPI) in an antifade solution. Each tissue core was evaluated for each FISH probe by manually counting signals in 20–60 intact non-overlapping interphase nuclei, using a fluorescence microscope (Leica DM6000 B). Manual scoring (Data Citation 3) was performed in tumour areas with loss of *PTEN* signals. The average of two experienced pathologists’ manual, independent assessment led to the final score. Two scoring methods were used: the percentage of aberrant nuclei and the ratio of *PTEN* to CEP10 signals. As a threshold for *PTEN* deletion, the percentage of aberrant nuclei was used in accordance to a previous publication^18^: hemizygous *PTEN* deletion was defined as the presence of fewer *PTEN* signals than CEP10 signals in at least 60% of counted nuclei. Homozygous *PTEN* deletion was defined if at least one third (33%) of aberrant nuclei revealed zero *PTEN* signal in a tissue core, with the presence of one or two *PTEN* signals in adjacent normal cells. Accordingly, *PTEN* deletion was defined if the average ratio of *PTEN* to CEP10 signals was less than or equal to 60%.

### *PTEN* DISH analysis

A BenchMark ULTRA automated stainer was used for the optimization and performance evaluation of the DISH assay for CEP10 and *PTEN* DNA targets. In this assay, a black signal represented the *PTEN* probe, a red signal corresponded to the CEP10, which were visualized with ultraView SISH DNP and Red ISH DIG detection kit respectively, after hybridization with the *PTEN* DNP probe and CEP10 probe cocktail. All tissue sections were counterstained with hematoxylin II and bluing reagent (Ventana). Air-dried glass slides were coverslipped using the Tissue-Tek Film automated coverslipper (Sakura Finetek Japan). The threshold of 60% for the ratio was used.

### Image-based computational workflow (ISHProfiler)

Tissue cores or slides were digitized and pre-processed (white balancing, deconvolution, and contrast modification) using the scanner’s default auto-correction settings. Images were then resized by bicubic interpolation to 4,096×4,096 pixels for efficient tiling (4,096=2^12^) and served as input data for the computational workflow. Pseudocode of the workflow was provided in the Supplementary Information of our related study^6^.

The basic workflow consists of three major algorithmic steps: First, each tissue was digitized, pre-processed, and resized. Second, DISH signals were detected by the circular Hough transform^19^. Third, a support vector machine (SVM) model^20^ was trained and validated (5-fold cross validation and grid search that iterates over all pairs of C and gamma) on an independent image set from a single tissue spot with the expert annotation, consisting of 1,000 image patches of size 13×13 pixels with *PTEN*, CEP10, *PTEN*+CEP10, white background noise and blue cell stains in the centre of the patch. The feature vector was constructed by concatenating (13×13=169) RGB values. The final model was used to classify the signals into five classes: *PTEN*, CEP10, mixed classes *PTEN*+CEP10, background noise and cell stains.

For reduction of misclassified signals, only gene and corresponding CEP signals were used for subsequent calculation. Signals classified as white or blue were discarded. The maximum of the global ratio was set to three to circumvent false positive gene signals due to unspecific staining (any roundish black signals) for cases with gene deletion. About 30% of signals were classified as *PTEN* or CEP10. Analogous to the ratio scoring method, the global ratio was defined as the division of all *PTEN* by all CEP10 signals in a single tissue core.

For the circular Hough transform, the signal radii were defined empirically from 1 to 7 pixels according to domain knowledge and the edge gradient threshold was set to Matlab default (Otsu's method). The detection sensitivity was set to Matlab default (0.85) for tissues scanned by the Zeiss scanner and was set to 0.95 for tissues digitized by the Hamamatsu scanner, because the Zeiss scanner has a higher image resolution and a more advanced sensor.

Based on the class and position of detected molecular signals, a signal colour map can be generated to visualize heterogeneous *PTEN* deletion. Moreover, advanced algorithms can be used for quantifying genetic alteration and investigating tumour heterogeneity^6^.

The computational workflow was implemented in Matlab (R2014b) and tested on a MacPro (2014). Matlab built-in functions for the circular Hough transform (*imfindcircles*) and ROC analysis (*perfcurve*) were used. The software package LIBSVM^21^ (version 3.18) was used to train, validate and test SVM models on the data.

### Code availability

The Matlab demo codes with initial parameter settings can be downloaded from https://github.com/zhoqi/SD_ISHProfiler. A basic workflow that classifies *PTEN* and CEP10 DISH signals has been integrated into the open source software TMARKER^22^ as a JAVA plug-in and is available at: http://www.nexus.ethz.ch/equipment_tools/software/tmarker/Plugins.html. In addition, R codes for survival analysis with Kaplan-Meier estimator and univariate and multivariate Cox regression are also openly available at: https://github.com/zhoqi/SD_survival.

## Data Records

### Image data

An image dataset (JPEG file format) of the *PTEN* DISH assay of 71 prostate cancer tissue samples, which were digitized by a Carl Zeiss Axio Scan.Z1 scanner, with a scanning resolution of 40x (0.11 μm per pixel). The native image resolution is 7,000×7,000 pixels for a single tissue core. Each tissue core is named as *_PTEN_Zeiss_7000.jpg, where * stands for the sample ID. A single tissue core as used in our related work^6^ has an image resolution of 4,096×4,096 pixels, which is named as *_PTEN_Zeiss_4096.jpg (Data Citation 1). Detailed technical specification of the scanner of the Zeiss scanner can be found at: http://applications.zeiss.com/C125792900358A3F/0/EC2B774E2EB1662AC1257ACB00506F68/$FILE/EN_41_011_023_Axio_Scan_Z1.pdf.

An image dataset (JPEG file format) of *PTEN* DISH assay of 71 prostate cancer tissue samples, which were digitized by a Hamamatsu C9600 NanoZoomer 2.0-HT Digital slide scanner, with a resolution of 40x (0.23 μm per pixel). The original image resolution is 3,100×3,100 pixels for a single tissue core, which is named as *_PTEN_Hamamatsu.jpg. In addition, 71 corresponding H&E images, named as *_HE_Hamamatsu.jpg, from serial sections were digitized by the same Hamamatsu scanner (Data Citation 2). Detailed technical specification of the Hamamatsu scanner can be found at: http://www.hamamatsu.com/resources/pdf/sys/SBIS0043E_NanoZoomers.pdf.

A whole tissue slide (108,000×138,000 pixels) of a transurethrally resected CRPC sample, which was hybridized with *PTEN* DISH and digitized with a Hamamatsu C9600 NanoZoomer 2.0-HT Digital slide scanner. The whole tissue side image (JPEG file format) was tiled into 3,726 single image patches of size 2,000×2,000 pixels, which were organized according to the accompanying README file and the overview image ‘B12-7405_8_boxes.jpg’ in PTEN.zip (Data Citation 4).

A second whole tissue slide (84,000×110,000 pixels) of an intra-ductal carcinoma of the prostate (IDC-P), which was hybridized with *PTEN* DISH and digitized with a Hamamatsu C9600 NanoZoomer 2.0-HT Digital slide scanner. The whole tissue side image (JPEG file format) was tiled into 2,310 single image patches of size 2,000×2,000 pixels, which were organized according to the accompanying README file and the overview image ‘B05-41379_10_boxes.jpg’ in PTEN_IDCP.zip (Data Citation 4).

Each tissue image was provided in its native.ndpi format, which can be handled by the OpenSlide (www.openslide.org) software. The.ndpi TMA DISH image contains all 71 images (PTEN_Hamamatsu.ndpi). The same applies to the.ndpi TMA H&E image (HE_Hamamatsu.ndpi). The two whole slide images (B12-7405_8.ndpi and B05-41379_10.ndpi) are provided in original resolution and not tiled into 2,000×2,000 pixels (Data Citation 5). Individual TMA cores in PTEN_Hamamatsu.ndpi and HE_Hamamatsu.ndpi are ordered as A_1_1, A_2_1, …, A_8_1, B_1_1, B_2_1, … B_8_1 from left to right, and A_1_1, A_1_2, …, A_1_13 from top to bottom.

### Clinical and survival data

Clinical and survival data of 424 prostate cancer patients (SPSS file format and tab-delimited format) with manual annotation of *PTEN* FISH for 339 RPE cases (Data Citation 3). The column names and their descriptions are listed in Table 2.

## Technical Validation

### Validated by independent images

The set of 71 DISH tissue cores was digitized by the bright-field and fluorescence slide scanner (Carl Zeiss) according to manufacturer’s instructions. In addition, we also performed a second digitization by using a Hamamatsu scanner. With application of the same parameter settings of circular Hough transform and SVM model, our image-based computational workflow produced similar classification results for both image datasets (r=0.83, *P*<0.0001), independent of scanning resolution and quality.

### Validation on two whole slide images

The whole slide image with 108,000×138,000 pixels was tiled into 3,726 image patches of size 2,000×2,000 pixels. We then used the same parameter settings as the TMAs for the calculation of circular Hough transform and the SVM model to detect and classify molecular signals. A landscape of *PTEN* deletion was generated by merging all signal colour maps of each image patches. The heterogeneous *PTEN* status matched closely to a serial section that was immunohistochemically stained with anti-PTEN antibody (Dako; clone 6H2.1)^6^. We performed the same validation on a second whole slide image with 84,000×110,000 pixels and obtained a similar result.

### Statistical validation of survival data

**Figure 2:**
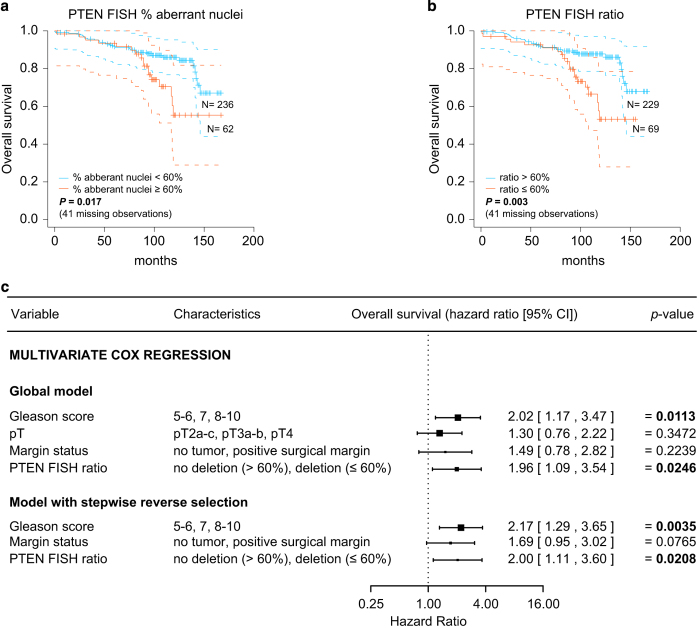
Survival analysis of 339 RPE patients. Kaplan-Meier curves with simultaneous 95% confidence bands of patient overall survival over time after diagnosis. Vertical lines illustrate patients who were censored at the time of their last clinical follow-up visit. Differences between survival estimates were evaluated by the log-rank test. *N* values represent the number of patients in each group under risk. For *P*-value, *P*<0.05 is marked in bold. (**a**) Kaplan-Meier curves for patients who were dichotomized into two groups by the percentage aberrant nuclei. (**b**) Kaplan-Meier curves for patients who were dichotomized into two groups by the ratio. (**c**) Overall survival hazard ratios by Cox regression. The dashed vertical line was drawn at the no effect point (hazard ratio of 1.0). Horizontal lines represent a 95% confidence interval (CI). The mid-point of the box represents the mean effect estimate and the area of the box represents the weight for each subgroup. Limit for the stepwise reverse selection procedure was *P*=0.1.

Nonparametric Kaplan-Meier estimators were used to analyse the overall, disease-specific and recurrence-free survival of the 339 RPE cases, of which 298 patients have median follow-up of 95 months with a variety of clinico-pathological, immunohistochemical and molecular features. We provided the original SPSS file (Data Citation 3) and R source codes (Code availability) for plotting a Kaplan-Meier curve for the overall survival (Fig. 2a,b) and for performing analysis of univariate and multivariable Cox regression (Fig. 2c), in which multivariate stepwise reverse selection was set to *P*=0.1 as the limit. The figures shown in this section are a modified version of Supplementary Figs 2 and 3 in our related work^6^, in which additional Kaplan-Meier curves for disease-specific and recurrence-free survival and forest plots for univariate Cox regression were reported.

## Usage Notes

### Brief instructions

The first dataset (Data Citation 1) comprises high quality images that were scanned by a Carl Zeiss Axio Scan.Z1 scanner. The leading part of the filename (e.g., A_1_1 from A_1_1 _PTEN_Zeiss_4096) matches the corresponding identifier in the first column of the annotation file demo1.xlsx (Loc: TMA core location; label: No deletion versus deletion; type: tissue type including BHP, RPE, CRPC, LNM, and DM; DISH_Manual: manual assessment of *PTEN* to CEP10 ratio).

To use ISHProfiler software, perform the following tasks.

1. Download the Matlab code from https://github.com/zhoqi/SD_ISHProfiler.
2. Install LIBSVM, version 3.18 or above, in the same directory as demo1.m.
3. Download the 71 images, named as *_PTEN_Zeiss_4096.jpg from (Data Citation 1), and place them in the directory ‘/images’.
4. Run demo1.m, demo2.m and demo3.m.

The file demo1.m outputs the global ratios for all 71 tissue cores. It also plots a sample tissue core image, superimposed with detected and classified *PTEN* gene and CEP signals. The *PTEN* signals are in black, CEP10 signals in red, and the mixed classes of *PTEN*+CEP10 signals in green. The file demo2.m plots pairwise distances of 71 tissue cores in the two-dimensional principal component analysis subspace, color-coded by *PTEN* DISH expert annotation. The file demo3.m plots six sample signal colour maps and outputs a computational model that specifies the position and colour of the detected molecular signals for each tissue core. With modifications of demo2.m, users can apply the code to the other datasets (Data Citations 2 and 4), without changing the pre-trained SVM model model.mat. For computational scientists, they can explore these rich datasets by providing their own user annotations or perform unsupervised learning of genetic alteration and tumour heterogeneity.

Survival analysis of the 339 RPE prostate cancer patients recorded in the SPSS file (Data Citation 3) can be performed by running the R code *pten_surv.R* (Code availability), which generates plots as in (Fig. 2) and in our related work^6^. Make sure that the external packages *survival*, *OIsurv* and *foreign* are pre-installed.

We are going to prepare a series of multi-omics (genomics, transcriptomics and proteomics) datasets, which are based on the same patient cohort of this manuscript. Therefore, the clinical and survival data of 424 prostate cancer patients serves as a master file for future reference (Data Citation 3).

### Access controls

Access permission to data and computer codes will be granted for research purpose only. Any interested researcher who will use the clinical survival data (Data Citation 3) is required to agree to the terms and conditions given by the authors prior to data access.

## Additional Information

**How to cite this article:** Zhong, Q. *et al.* A curated collection of tissue microarray images and clinical outcome data of prostate cancer patients. *Sci. Data* 4:170014 doi: 10.1038/sdata.2017.14 (2017).

**Publisher’s note:** Springer Nature remains neutral with regard to jurisdictional claims in published maps and institutional affiliations.

## Supplementary Material



## Figures and Tables

**Table 1:** Summary of DISH images, *PTEN* genetic status, clinical information and survival data of patient cohorts with prostate cancer.

**Subjects**	**Protocol 1**	**Protocol 2**	**Protocol 3**	**Protocol 4**	**Data**
Prostate cancer TMA (*n*=71)	DISH staining and digitization by Zeiss	Manual assessment	Computational analysis (Matlab codes)	Comparison with FISH and patient-level annotation	Data Citation 1
Prostate cancer TMA (*n*=71)	DISH and H&E staining and digitization by Hamamatsu	Manual assessment	Computational analysis (Matlab codes)	Comparison with FISH and patient-level annotation	Data Citation 2
Prostate cancer (*n*=424)	FISH staining, collection of clinical and survival data	Manual assessment	Kaplan-Meier estimator (R codes)	Univariate and multivariate Cox regression	Data Citation 3
Prostate cancer and two whole slide images (*n*=3,726; *n*=2,310)	DISH staining and digitization	NA	Computational analysis	Post visual inspection	Data Citation 4
Prostate cancer TMA (*n*=71) and two whole slide images (*n*=3,726; *n*=2,310)	DISH and H&E staining and digitization by Hamamatsu	NA	NA	Output.ndpi file format	Data Citation 5

**Table 2:** Summary of clinical and survival data.

**Variable Name**	**Description (range or categorization)**
TMA	References the ‘Loc’ variable in demo1.xlsx (ISHProfiler^®^) (e.g., A_1_1)
rfs	Recurrence-free survival (0–163 months)
st_rfs	Status for recurrence-free survival (0: censored, 1: recurrence, 3: never reached nadir)
os	Overall survival (0–167months)
st_os_gen	Status for overall survival (0: censored, 1: any death)
st_os_spec	Status for disease-specific survival (0: censored, 1: death from prostate cancer)
tiss	Tissue type (1: RPE, 2: CRPC, 3: LNM, 4: DM, 8: BPH)
age_d64	Age at RPE (dichotomization by 64) (0<64 years, 1≥64 years)
BMI_d25	Body mass index (dichotomization by 25) (0≤25 kgm^−2^, 1>25 kgm^−2^)
GLE_t	Gleason score (1: Gleason score 5–6, 2: 7, 3: 8–10)
pT_t	Tumour stage (1: pT2, 2: pT3, 3: pT4)
pN	Nodal status (0: no tumour, 1: positive surgical margin)
R1	Surgical margin status (0: R0, 1: R1)
PSA_d10	PSA at diagnosis (dichotomization by 10) (0<10 ngml^−1^, 1≥10 ngml^−1^)
PTEN_cyt	PTEN IHC (cytoplasmic intensity) (0: negative, 1: weak, 2: moderate, 3: strong)
SPOP	SPOP mutation FD (0: wild-type, 1: mutated)
ERG	ERG break-apart FISH (0: FISH−, 1: FISH+)
PTEN_FISH_ratio	PTEN FISH ratio (0.28–1.00)
PTEN_FISH_ratio_d60	PTEN FISH ratio (dichotomization by 0.6) (0: no deletion (>0.6), 1: PTEN deletion (≤0.6))
PTEN_FISH_percent_ab_nucl	PTEN FISH % aberrant nuclei (0–1.00)
PTEN_FISH_percent_d60	PTEN FISH % aberrant nuclei (dichotomization by 0.6) (0: no deletion (<60%), 1: PTEN deletion (≥60%))
PTEN_total_signals	Total PTEN FISH signals (10–123)
nuclei_number	Number of nuclei analysed (9–40)
CEP10_total_signals	Total CEP10 FISH signals (17–127)

## References

[d1] Harvard Dataverse Zhong Q. Wild P. 2016 http://dx.doi.org/10.7910/DVN/RRKMHC

[d2] Harvard Dataverse Zhong Q. Wild P. 2016 http://dx.doi.org/10.7910/DVN/4WEMEQ

[d3] Harvard Dataverse Zhong Q. Wild P. 2016 http://dx.doi.org/10.7910/DVN/01KX3V

[d4] Harvard Dataverse Zhong Q. Wild P. 2016 http://dx.doi.org/10.7910/DVN/KT4WSK

[d5] Harvard Dataverse Zhong Q. Wild P. 2016 http://dx.doi.org/10.7910/DVN/GG0D7G

